# Engineering *Saccharomyces cerevisiae* for the production of the valuable monoterpene ester geranyl acetate

**DOI:** 10.1186/s12934-018-0930-y

**Published:** 2018-06-05

**Authors:** Tao Wu, Siwei Li, Bolin Zhang, Changhao Bi, Xueli Zhang

**Affiliations:** 1 College of Biological Sciences and Technology, Beijing Forestry University, Beijing, 100083 People’s Republic of China; 2 Tianjin Institute of Industrial Biotechnology, Chinese Academy of Sciences, Tianjin, 300308 People’s Republic of China; 3 Key Laboratory of Systems Microbial Biotechnology, Chinese Academy of Sciences, Tianjin, 300308 People’s Republic of China

**Keywords:** Monoterpene, Geranyl acetate, ERG20, tHMG1, IDI1, MAF1

## Abstract

**Background:**

Geranyl acetate is widely used in the fragrance and cosmetic industries, and thus has great economic value. However, plants naturally produce a mixture of hundreds of esters, and geranyl acetate is usually only present in trace amounts, which makes its economical extraction from plant sources practically impossible. As an ideal host for heterologous production of fragrance compound, the *Saccharomyces cerevisiae* has never been engineered to produce the esters, such as geranyl acetate.

**Results:**

In this study, a heterologous geranyl acetate synthesis pathway was constructed in *S. cerevisiae* for the first time, and a titer of 0.63 mg/L geranyl acetate was achieved. By expressing an Erg20 mutant to divert carbon flux from FPP to GPP, the geranyl acetate production increased to 2.64 mg/L. However, the expression of heterologous GPP had limited effect. The highest production of 13.27 mg/L geranyl acetate was achieved by additional integration and expression of tHMG1, IDI1 and MAF1. Furthermore, through optimizing fermentation conditions, the geranyl acetate titer increased to 22.49 mg/L.

**Conclusions:**

We constructed a monoterpene ester producing cell factory in *S. cerevisiae* for the first time, and demonstrated the great potential of this system for the heterologous production of a large group of economically important fragrance compounds.

**Electronic supplementary material:**

The online version of this article (10.1186/s12934-018-0930-y) contains supplementary material, which is available to authorized users.

## Background

Monoterpenes constitute a subclass of terpenoids [1] that are widely used as additives in the food, pharmaceutical, agrichemical and cosmetic industries, due to their strong flavor, fragrance and physiological activity [2, 3]. Moreover, some monoterpenes were shown to have great potential as biofuels [4], which prompted increased attention from the research community in recent years. The basic scaffold of monoterpenes contains two isoprene units that are biosynthesized from geranyl diphosphate (GPP), which in turn is synthesized from isopentenyl diphosphate (IPP) and its isomer dimethylallyl diphosphate (DMAPP), derived from either the mevalonate (MVA) pathway or the 2C-methyl-d-erythrtiol 4-phosphate (MEP) pathway [5, 6]. As the precursor of monoterpenes such as geraniol and linalool, GPP reacts with one more IPP and gives rise to farnesyl diphosphate (FPP) [7, 8], which is the precursor of sesquiterpenes, squalene, diterpenes, GGPP and so on.

Monoterpenes are mainly produced by plants, albeit at extremely low concentrations [9], and the traditional chemical synthesis and bio-extraction processes are both costly and environmentally harmful. However, a number of research groups have been able to produce natural products by metabolic engineering of microbial cell factories [10, 11], most often derived from the model organisms *Escherichia coli* and *Saccharomyces cerevisiae* [12–14]. The production of monoterpenes by metabolic engineering has also been reported, but most of the cases involved relative low production, which hindered their industrial application [9]. Previous work has shown that engineered *E. coli* could produce 400 mg/L of limonene and approximately 100 mg/L of perillyl alcohol [13], and engineered yeasts were able to produce 95 µg/L of linalool [15], as well as 36.04 mg/L–2.0 g/L of geraniol [16, 17].

**Fig. 1:**
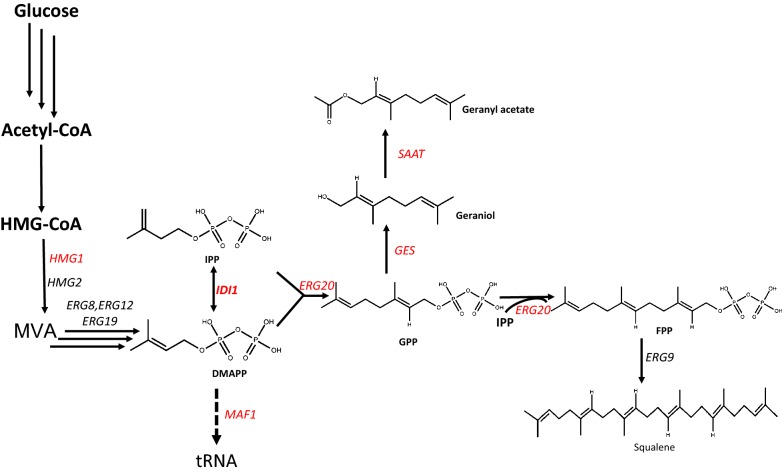
Schematic diagram of the heterologous synthesis pathway for the production of geranyl acetate in the engineered *S. cerevisiae*. *HMG-CoA* 3-hydroxy-3-methylglutaryl coenzyme A, *MVA* mevalonate, *IPP* isopentenyl pyrophosphate, *DMAPP* dimethylallyl diphosphate, *GPP* geranyl pyrophosphate, *FPP* farnesyl pyrophosphate

*Saccharomyces cerevisiae* possesses a native MVA pathway [6], which makes it suitable for the synthesis of monoterpenes. Some reports also demonstrated the potential of metabolic engineering for monoterpene production in *S. cerevisiae* [18]. GPP is synthesized by the bifunctional enzyme ERG20 (Fig. 1), which has both GPP synthase (GPPS) and farnesyl pyrophosphate synthase (FPPS) activities [19]. To decrease the metabolic flux towards FPP, mutants with changes in the FPP synthesis domain of Erg20p were screened, and mutations at position 197 (K197G, C, S, T, D, E) [18] and a double mutant (N127W–F96W) [20] were proved to dramatically increase the yield of monoterpenes. To increase the production of sabinene, the sabinene synthase SpSabS1 was fused to the Erg20 variant, and 1.87 mg/L sabinene production was achieved, which represents a 3.5-fold increase compared with that achieved via the separate expression of Erg20 and SpSabS1 [20]. By the screening of different sources of GESs and GPPSs, as well as fusions of the two proteins, Hou’ group achieved the titer of 293 mg/L geraniol in *S. cerevisiae* [21]. Furthermore, dynamic control of ERG20 expression combined with minimized endogenous downstream metabolism led to impressive progress in the production of geraniol [22].

On the other hand, HMG-CoA reductase was identified as a key rate-limiting enzyme in the MVA pathway of *S. cerevisiae* [23, 24], and a truncated 3-hydroxyl-3-methylglutaryl-CoA reductase gene (*tHMGR*) was overexpressed to increase the supply of mevalonate. DMAPP is the common substrate for the biosynthesis of both GPP and tRNA, and MAF1 represses the transcriptional activity of RNA polymerase III, serving as a negative regulator in the biosynthesis of tRNA [16]. The isoprenoid diphosphate isomerase IDI1 catalyzes the interconversion between DMAPP and IPP [25], but the formation of one molecule of GPP requires two molecules of IPP and one molecule of DMAPP. Since the ratio of IPP to DMAPP is not optimal for GPP biosynthesis, Jingwen Zhou’s research indicated that the isomerase IDI1 was the rate-limiting enzyme in geraniol production [16]. Thus, overexpression of truncated HMG-CoA (tHMG1), IDI1 and MAF1 could improve the production of monoterpenes.

Geranyl acetate, an acyclic monoterpene ester derived from geraniol, is widely used in the cosmetics industry due to its pleasant scent, and it was also recently discovered to have an antinociceptive activity [26], making it a compound with great economic value. However, plants naturally produce a mixture of hundreds of esters, and geranyl acetate only makes up a small percentage of the total, which makes its extraction and traditional plant-based production uneconomical [27, 28]. Albeit the great commercial potential, as far as we know, no research has focused to heterologous produce ester fragrance compounds. And as an ideal host for heterologous production of fragrance compound, the *S. cerevisiae* has never been engineered to produce the esters, such as geranyl acetate.

Thus, in this study, we intended to construct a *S. cerevisiae* cell factory for production of geranyl acetate to study and demonstrate the capacity of this system for heterologous production of this group of economically important fragrance compounds, and explore the fermentation methods and conditions for the production process (Fig. 1). Geraniol synthase (GES) [16, 22, 29] from *Ocimum basilicum* and alcohol acyltransferases (AATs) [29] from strawberry (*Fragaria *×* ananassa*) (SAAT) were introduced into the yeast chromosome. Several groups have used the GES from *O. basilicum* for the formation of geraniol, and SAAT showed a high affinity and efficiency for the biosynthesis of geranyl acetate [29].

## Methods

### Media, strains and plasmids

*Escherichia coli* Trans T1 (TransGen, China) was used as the host for plasmid construction and amplification. The cells were grown at 37 °C in Luria–Bertani (LB) medium (1% NaCl, 1% tryptone and 0.5% yeast extract with 100 mg/L of ampicillin (Solarbio, China). *S. cerevisiae* BY4742 (*MATα, his3Δ1, leu2Δ0, lys2Δ0, MET15, ura3Δ0*; Euroscarf, Germany) was used as the host strain for DNA assembly and integration. The cells were cultivated at 30 °C in yeast-extract peptone dextrose (YPD) medium (2% glucose, 2% peptone, and 1% yeast extract). Synthetic complete drop-out medium lacking leucine and/or histidine (SC-LEU, SC-LEU-HIS, SC-LEU-HIS-TRP) was used for transformant selection. For solid media, 2% agar was added.

The codon-optimized genes encoding geraniol synthase (GES) from *Ocimum basilicum* without the N-terminal transit peptide (1–34aa), and alcohol acyltransferases (AATs) (SAAT) from cultivated strawberry (*Fragaria *×* ananassa*) were synthesized by GENEWIZ (Suzhou, China) and embedded in plasmid pUC57-Amp. The sequences of the two genes are provided in Additional file 1. The SAAT and GES genes were excised from pUC57-SAAT/GES with *Sex*AI and *Asc*I. *SAAT* was ligated into the vector pM2 under the control of the *PGK1* promoter and *ADH1* terminator, and pM3-GES was constructed with GES under the control of the *TEF2* promoter and *CYC1* terminator. *tHMG1* containing the catalytic domain of HMG1 was amplified from the genome of *S. cerevisiae* using the primer pair *SexA*-*tHMG1/AscI*-*tHMG1* and cloned into vector pM4 using T4 ligase (Thermo, USA). The plasmids pM4-ERG20(F96W–N127W), pM2-MAF1 and pM3-IDI1 were constructed in the same way (Table 1).

**Table 1:** Strains and plasmids used in this study

Name	Description	Source
pUC57-GES	Cloning vector with a synthetic version of the GES gene from *O. basilicum*	GENEWIZ
pUC57-SAAT	Cloning vector with a synthetic version of the SAAT gene from cultivated strawberry (*Fragaria *× *ananassa*)	GENEWIZ
pEASY-Blunt	Cloning vector for blunt ligation	This study
pM3-GES	pEASY-Blunt vector with pTEF2-GES-tCYC1cassette	This study
pM2-SAAT	pEASY-Blunt vector with pPGK1-SAAT-tADH1 cassette	This study
pM4-ERG20(F96W–N127W)	pEASY-Blunt vector with pTDH3-ERG20(F96W–N127W)-tTPI1 cassette	This study
pM2-MAF1	pEASY-Blunt vector with pPGK1-MAF1-tADH1 cassette	This study
pM3-IDI1	pEASY-Blunt vector with pTEF2-IDI1-tCYC1cassette	This study
pM4-tHMG1	pEASY-Blunt vector with pTDH3-tHMG1-tTPI1 cassette	This study
pRS313-TRP	pRS313 vector: HIS selection marker was replased withTRP	This study
pRS313-GPPS_At_	pRS313-TRP vector with pPGK-GPPS_At_-tCYC1cassette	This study
pRS313-GPPS_Mp_	pRS313-TRP vector with pPGK-GPPS_Mp_-tCYC1cassette	This study

The plant GPP synthase genes were amplified from cDNA of *Arabidopsis thaliana* and *Mentha piperita*, and cloned into the expression vector pRS313-TRP using T4 ligase. The maps of the corresponding expression vectors pRS313-GPPS_At_ and pRS313-GPPS_Mp_ are shown in Additional file 1: Fig. S1. All plasmids were verified by PCR and DNA sequencing, and the relevant primers are listed in Additional file 1: Table S1.

### Site-directed Mutagenesis of the farnesyl diphosphate synthase *ERG20*

Two point-mutations (F96W and N127W) were introduced into ERG20 by overlap-extension PCR. Yeast genomic DNA was used as the template, and the ERG20 gene was divided into three parts, 1–96aa, 94–127aa and 127–353aa. The three parts of the gene were amplified separately using primers with embedded mutations, and then fused into a complete mutated gene by overlap-extension PCR. The corresponding primers are listed in Additional file 1: Table S1.

### Genetic manipulation of *S. cerevisiae*

**Fig. 2:**
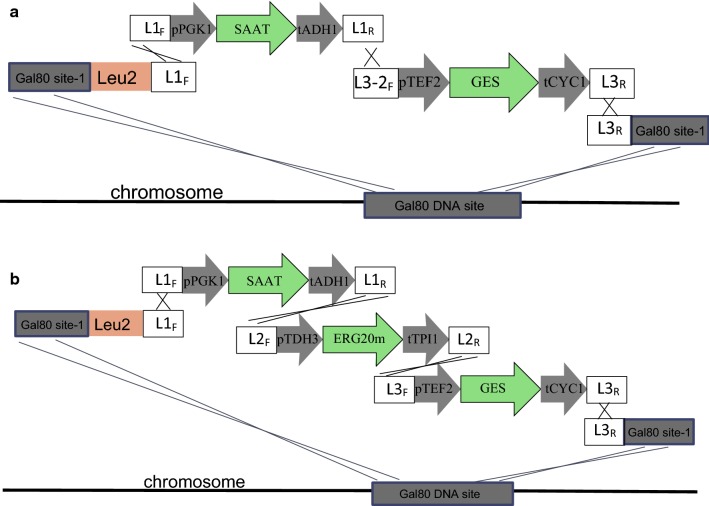
Schematic diagram of the integrated expression cassettes of the heterologous geranyl acetate synthesis pathway (**a**) the yeast genome integration of geraniol synthase gene (GES) and alcohol acyltransferases gene (SAAT) (**b**) the yeast genome integration of the geraniol synthase gene (GES), strawberry acyltransferases gene (SAAT) and Erg20 mutant (F96W–N127W) gene

The construction and assembly of functional transcriptional units on the chromosome of *S. cerevisiae* was performed by the DNA assembler method [30, 31]. The transcription unit encoding GES (*pTEF2*-*GES*-*tCYC1*) was amplified by PCR from plasmid pM2-GES, that of SAAT (*pPGK1*-*GES*-*tADH1*) from pM3-SAAT, and that of ERG20 (*pTDH3*-*ERG20(F96W*–*N127W)*-*tTPI1*) from pM4-ERG20. The selection marker and integration locus fragments were amplified by PCR. All primers used for amplification and integration into the *S. cerevisiae* chromosome are listed in Additional file 1: Table S1. Strain GA01 was constructed by integrating the GES and SAAT cassettes into the *gal 80* site along with the LEU selection marker, the strain GA02 was constructed by integrating the GES, SAAT and ERG20(F96W–N127W) cassettes into the *gal 80* site along with the LEU selection maker (Fig. 2), and strain GA03 was constructed by integrating the tHMG1, MAF1 and IDI1 cassettes into the *NDT80* site of strain GA02 along with the HIS selection maker.

The DNA fragments, GPP synthase expression plasmid pRS313-GPPSAt and pRS313-GPPSMp were all introduced into *S. cerevisiae* BY4742 by conventional electroporation method. When four or five fragments (each fragment was 100 ng) were used for homologous recombination (HR) in *S. cerevisiae*, about 200–400 colonies could be achieved.

### Yeast cultivation and PCR confirmation

For PCR confirmation of transformants, single colonies were used to inoculate 4 mL of SC-LEU/SC-LEU-HIS/SC–LEU-HIS-TRP medium, and grown at 30°C and 250 rpm overnight. Cells were harvested by centrifugation, and the genomic DNA was extracted using the Yeast Gene DNA Kit (CW Biotech, China). 2 μL of total DNA was used as template for PCR using the 2 × Taq Master Mix (CW Biotech). 10 colonies from SC-Leu agar plates were randomly picked and inoculated in SC-Leu medium. After that, genomic DNA was extracted, and PCR determination was performed respectively. The ratio of positive clones to all the colonies was calculated to be above 90%.

### Cell-culture, extraction and quantification of geranyl acetate

The correct colonies were picked and grown in the corresponding synthetic complete drop-out medium or YPD medium overnight, transferred into a flask with fresh medium to yield an initial OD_600_ of 0.05–0.10, and cultured for 6 days at 30 °C and 250 rpm. The increase of cell biomass during the fermentation process was detected by measuring the OD_600_ value using a UV-2550 spectrophotometer (Shimadzu, Japan).

To quantify the titer of geranyl acetate in the yeast cultures, aliquots comprising 1 mL of the fermentation broth were concentrated by centrifugation at 16,200×*g* for 2 min, after which 1 mL of *n*-hexane was added to extract the products that were secreted into the medium. The cell pellet was extracted with another 1 mL of n-hexane under ultrasonic agitation for 30 min, and the n-hexane phase was collected by centrifugation at 16,200×*g* for 2 min. The two extraction liquids were mixed, and 1 µL of the combined extract was analyzed using a Agilent 5975C GC–MS system equipped with a HP-5 ms GC column (30 m × 0.25 mm × 0.5 μm; Agilent, USA) and a triple-Axis detector. The GC–MS temperature program encompassed an initial temperature of 45°C for 1 min and a ramp of 10°C/min to 220°C, which was maintained for 5 min. Helium was used as the mobile phase at a flow rate of 1.0 mL/min. The injection port, interface, and MS source temperatures were 250, 300, and 180 °C, respectively [29]. A reference standard comprising authentic geranyl acetate purchased from Sigma Aldrich, USA, was used for quantification.

## Results

### Construction of a microbial cell factory by integrating the geranyl acetate biosynthetic pathway into the chromosome of *S. cerevisiae*

As volatile esters, geranyl acetate is the essential components of fruit characteristic aroma and presents in the essential oils of various plant species. It serves as responser to stress or insect infestation. It has been reported that truncated *O. basilicum* geraniol synthase is very efficient in geraniol synthesis with geranyl diphosphate (GPP) as the substrate [3, 16]. We found that alcohol acyltransferase gene from cultivated strawberry (SAAT) was a highly active enzyme capable of transferring the acetyl group from acetyl-CoA to various substrates, and possibly to geraniol to form geranyl acetate [29]. In order to construct a metabolic pathway for the production of geranyl acetate, the geraniol synthase (GES) gene from *O. basilicum* and SAAT were integrated into the chromosome of *S. cerevisiae* BY4742 at the *gal80* site (Fig. 2a). The expression of the synthetic cassette was controlled by a constitutive strong promoter and the resulting strain was designated as GA01.

**Fig. 3:**
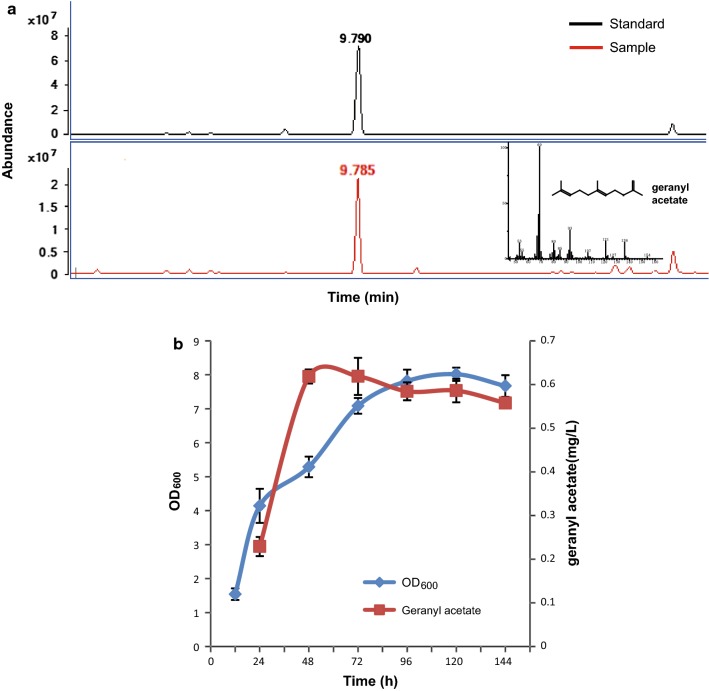
Identification of fermentation products of strain GA01. **a** The time-course of OD_600_ and geranyl acetate in aerobic fermentation of strain GA01. 38. **b** GC–MS analysis of a cell extract of strain GA01. The mass spectrum of geranyl acetate is shown in the top right corner and the red line indicates the authentic geranyl acetate standard

The production of geranyl acetate was measured by GC–MS (Fig. 3a), and the titer ranged from 0.25 to 0.63 mg/L during the fermentation process (Fig. 3b). The titer reached its maximum value after 48 h of cultivation, at an OD_600_ of 5.23. However, while the density of the yeast culture increased persistently for 5 days, geranyl acetate production did not improve accordingly, which may be due to gaseous escape of the volatile geranyl acetate during the aerobic fermentation process [32]. Besides, another four alcohol acyltransferase genes from plants were used for geranyl acetate production, but the titer was very low or no geranyl acetate was detected compared with SAAT (Additional file 1: Table S2).

### The expression of Erg20 mutants for improved geranyl acetate production

**Fig. 4:**
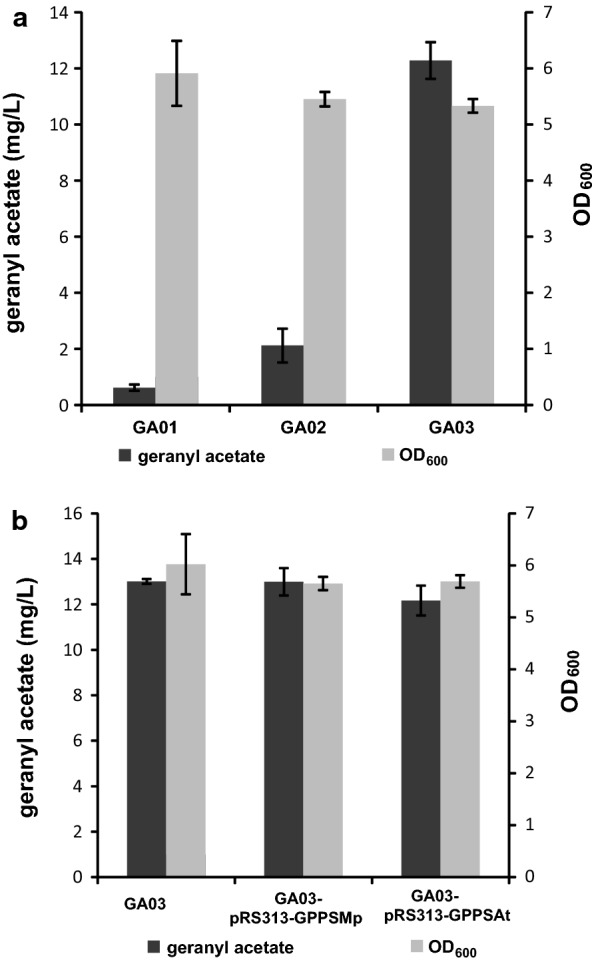
Fermentation and quantification of geranyl acetate. **a** OD_600_ and geranyl acetate titer of the engineered strains GA01, GA02 and GA03. **b** Geranyl acetate titers of strains with plant GPP synthases (GPPS_At_: *Arabidopsis thaliana* GPP synthase, GPPS_Mp_: *Mentha piperita* GPP synthase)

GPP is the starting substrate of monoterpene production pathways [19]. However, in yeast cells GPP can be converted into FPP by the bifunctional synthase Erg20. In addition, since FPP is the precursor of ergosterol, the outright deletion of Erg20 is potentially lethal [5]. Accordingly, the Erg20 F96W–N127W mutant, which has a decreased FPP formation efficiency but consistent GPP production, was constructed according to an earlier report [20]. The mutated Erg20, GES and SAAT genes were integrated into the chromosome of *S. cerevisiae* BY4742 at the *gal80* site (Fig. 2b). The resulting strain GA02 produced 2.64 mg/L of geranyl acetate in 48 h, which represented a 419% increase of titer over GA01 (Fig. 4a).

### Overexpression of tHMG1, IDI1 and MAF1 for the enhanced production of geranyl acetate

Isoprenoid diphosphate isomerase (IDI1) catalyzes the isomeric interconversion between IPP and DMAPP [33], and its overexpression can therefore potentially enhance the synthesis of GPP and benefit the production of monoterpenes. MAF1 represses the transcriptional activity of RNA polymerase III, serving as a negative regulator of the biosynthesis of tRNAs [16, 18]. Since DMAPP is a common substrate of both tRNA and GPP synthesis, overexpression of MAF1 can divert the carbon flux toward GPP formation [18]. Thus, to further improve the biosynthesis of geranyl acetate, IDI1, MAF1 and tHMG1 were integrated into the chromosome of GA02, resulting in strain GA03. In this best strain, the titer of geranyl acetate reached 13.27 mg/L, representing a 400% increase compared with GA02, and a remarkable 2100% increase over the starting strain GA01 (Fig. 4a).

Since *S. cerevisiae* does not have a specific GPP synthase (GPPS, EC 2.5.1.1), which belongs to the short-chain prenyltransferase family [34]. To supply more precursor for GPP synthesis, The homomeric GPPS from *Arabidopsis thaliana* (GPPS_At_) and the heteromeric GPPS from *Mentha piperita* (GPPS_Mp_) were separately introduced into GA03 on the plasmid pRS313-TRP, resulting in the strains GA03-pRS313-GPPS_At_ and GA03-pRS313-GPPS_Mp_ respectively. The relevant primers are listed in Additional file 1: Table S1, and plasmid diagrams are shown in Additional file 1: Fig. S1. Structurally, the heteromeric GPPS is composed of a large subunit (LSU) and a small subunit (SSU) [19]. The LSU is inactive alone, and the non-catalytic SSU acts as a modulator of the interaction between the two inactive subunits, resulting in an active GPPS [19]. Consequently, the flexible fusion protein linker GGGS and (GGGS)_3_ were introduced to construct a heteromeric *Mentha piperita* GPPS (LSU-GGGS/(GGGS)_3_-SSU). Unfortunately, the introduction of plant GPPS did not effectively improve the production of geranyl acetate (Fig. 4b). Since fusion of the two subunits might affect the expression and function of GPPS protein, we constructed another plasmid pRS313-pPGK-LSU-tCYC-pTEF-SSU-tADH,the two subunit were expressed with strong promoter respectively, but the geranyl acetate production was not increased either.

### Improving geranyl acetate production by optimizing the fermentation conditions

**Fig. 5:**
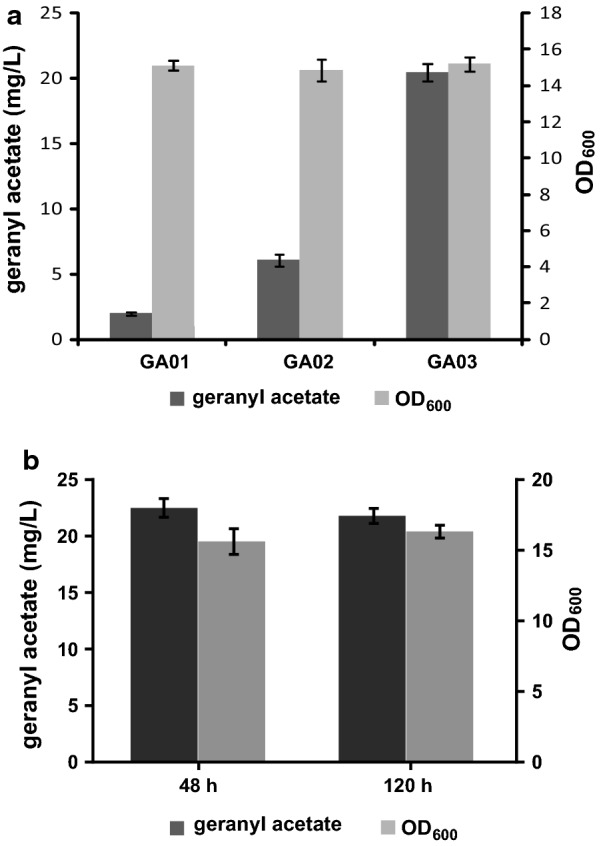
Fermentation optimization and titer of geranyl acetate. **a** OD_600_ and geranyl acetate titer of the engineered strains GA01, GA02 and GA03 in YPD medium. **b** Geranyl acetate titer of strains in YPD medium with 10% isopropyl myristate added to the culture after 24 h

The highest geranyl acetate production of strain GA3 reached to 13.27 mg/L in SC-LEU-HIS medium after cultivated for 48 h, and the OD_600_ reached about 5.23 (Fig. 4a). To further improve geranyl acetate production, we adjusted the initial OD_600_ of fermentation to 0.1, which made the final OD_600_ increased to about 7.2 in synthetic complete drop-out medium, and to about 15.6 in YPD medium after incubated at 30 °C for 48 h. When YPD medium was used to fermentation, the titer of geranyl acetate increased to 1.98, 6.07 and 20.48 mg/L respectively for strain GA01, GA02 and GA03 after cultivating for 48 h respectively, as shown in Fig. 5a. Furthermore, in order to prevent volatilization of geranyl acetate, 10% isopropyl myristate was added to the culture medium after 24 h. The production of geranyl acetate increased to 22.49 mg/L in strain GA03 as shown in Fig. 5b, and the organic layer was easily harvested by centrifugation of the fermentation medium. However, we found geranyl acetate production did not improve continually when the strains were inoculated for 5 days. The geranyl acetate production was improved by 1.69-fold through optimization of the fermentation conditions.

## Discussion

In the past decades, more and more attention has arisen on heterologous production of the monoterpene geraniol. The highest titer of geraniol is about 2.0 g/L in engineered *E. coli* and 1.68 g/L in engineered *S. cerevisiae* respectively [17, 22]. However, there are rare reports for metabolic engineering of heterologous production of monoterpene esters, such as geranyl acetate. Monoterpenoids such as geranyl acetate are active compounds derived from many plants, which play important roles in protection against pathogens and attraction of animals, also traditionally used as additive of medicines, essential oils and perfume [26].

The truncated *O. basilicum* geraniol synthase (GES) and alcohol acyltransferase from strawberry (SAAT) were found with high activity in our lab [22, 29], the two genes were integrated into the chromosome of *S. cerevisiae* BY4742 at the *gal80* site (Fig. 2a) and a titer of 0.63 mg/L was achieved. According to the method of Huizhou Liu’s group [17], the titer of geraniol in culture medium and in yeast cells after fermentation was analyzed, which was measured to be zero. This result indicated geraniol was entirely converted to geranyl acetate by SAAT by the strains we constructed, and suggested the SAAT we used was very efficient and not the rate-limiting step of the synthesis pathway. Besides, a report just published described truncated GES from *Catharanthus roseus* with site-directed mutation (Y436 and D501) was found to has high catalytic activity [35]. Thus, for the further improvement of the geranyl acetate production, selection and optimization of GESs might be the key focus. In engineered *S. cerevisiae* producing monoterpene, the farnesyl diphosphate synthase Erg20p was found to be the key enzyme that limiting monoterpene formation [20]. GPP and FPP formation are both catalyzed by Erg20p, towards either geraniol or downstream squalene synthesis. For more GPP synthesis, Erg20 F96W–N127W mutant was integrated into the genome, which had decreased farnesyl diphosphate synthase function without interference of the growth of *S. cerevisiae*. And the production of geranyl acetate increased to 2.64 mg/L. Furthermore, a higher production of 13.27 mg/L was achieved by additional integration and expression of tHMG1, IDI1 and MAF1, Overexpression of which with strong promoters increased the supplement of precursors.

In the fermentation optimization process, when we used YPD media instead of the synthetic complete drop-out medium for fermentation, the OD_600_ value of the fermentation broth and the yield of geranyl acetate were both increased. The OD_600_ value could reach up to about 15.6, presenting a 198% increase compared with that in SC-LEU-HIS medium. The titer of geranyl acetate reached 20.48 mg/L, showing a 54% increase compared with the production in SC-LEU-HIS medium (13.27 mg/L). So YPD was more suitable for cell growth and fermentation of the engineered strains. Besides,due to the possible gaseous escape of the volatile geranyl acetate, 10% isopropyl myristate was added to the culture after 24 h fermentation. TThe production of geranyl acetate increased to 22.49 mg/L in strain GA03 with YPD medium as shown in Fig. 5b. However, geranyl acetate production did not improve continually in the 5-days fermentation process. We thought during the time, the accumulation of the product might affect the growth condition and inhibit further improvement of geranyl acetate production as described by Zhao’s article [21].

To sum up, optimization of the fermentation conditions led to a 1.69-fold improvement of geranyl acetate production. And with 10% isopropyl myristate added, it might be able to prevent the volatilization of geranyl acetate and relieve the cell toxicity of geranyl acetate by extracting it from the fermentation broth [21].

## Conclusion

**Fig. 6:**
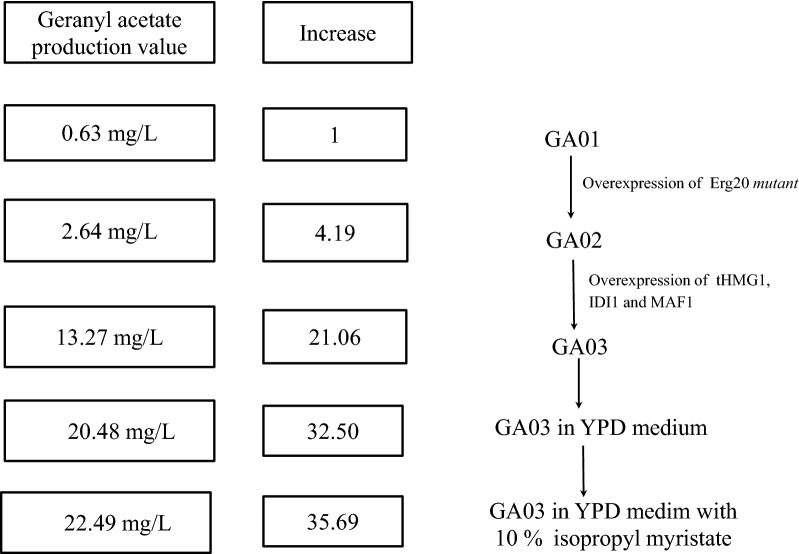
Diagram summarizing the increase of geranyl acetate

In this research, a heterologous geranyl acetate synthesis pathway was constructed in *S. cerevisiae* for the first time, and a product titer of 0.63 mg/L was achieved in the starting strain. By expressing an Erg20 mutant to divert carbon flux from FPP to GPP, the geranyl acetate production was increased to 2.64 mg/L, although expression of heterologous GPP did not have a significant effect. The highest production of 13.27 mg/L was achieved by additionally expressing tHMG1, IDI1 and MAF1, which represents a remarkable 2100% increase over the starting strain. Furthermore, optimization of the fermentation conditions led to 22.49 mg/L geranyl acetate production, which exhibited a 35.69-fold increase over the parent strain GA01 (Fig. 6). To our best knowledge, this work offers the first microbial cell factory for specific production of a monoterpene ester, demonstrating great potential for the heterologous production of many more economically important fragrance compounds.

## Additional file

**Additional file 1: Table S1.** Primers used in this study. **Table S2.** Introduction of alcohol acyltransferases from plants and the titer of geranyl acetate. **Fig. S1.** Plasmid maps and DNA sequences.
