# Supplementary material for: Engineering Saccharomyces cerevisiae for the production of the valuable monoterpene ester geranyl acetate
Source: Microb Cell Fact. 2018 Jun 5;17:85. doi: 10.1186/s12934-018-0930-y (PMC5987629; doi:10.1186/s12934-018-0930-y)
Supplement: Supplementary file 1 — Additional file 1: Table S1. Primers used in this study. Table S2. Introduction of alcohol acyltransferases from plants and the titer of geranyl acetate. Fig. S1. Plasmid maps and DNA sequences. [file 12934_2018_930_MOESM1_ESM.docx]

**Additional File 1**

**Table S1 The primers used in this research**

| Primers | Sequence (5′→ 3′) | Sources |
| --- | --- | --- |
| SexAI-GES | ACCTGGTTCTGCTTGTACTCCTTTG | This study |
| AscI-GES | GGCGCGCCTTATTGAGTG | This study |
| SexAI-SAAT | ACCTGGTATGGAAAAAATTGAAGTC | This study |
| AscI-SAAT | GGCGCGCCTTAAATCAATG | This study |
| SexAI-ERG20 | GCACCTGGTATGGCTTCAGAAAAAGAAATTAG | This study |
| AscI-ERG20 | CTGGCGCGCCCTATTTGCTTCTCTTGTAAACTTTG | This study |
| 1-M-pEASY-PGK1-F | CTGTTTCCTGTGTGAAATTGTTATCCGCTCACAATTCCACACAACATACGAGCCTTAATTAAACGCACAGATATTATAAC | This study |
| 3G-1-M-ADHT-TDH3-R | CCTCCGCGTCATTAAACTTCTTGTTGTTGACGCTAACATCAACGCTAGTATTCGGCATGCCGGTAGAGGTGTGG | This study |
| 3G-3-M-ADH1t-TDH3-F | CAGGTATAGCATGAGGTCGCTCTTATTGACCACACCTCTACCGGCATGCCGAATACTAGCGTTGAATGTTAGCGTC | This study |
| 3G-3-M-TPL1t-TEF1-R | AGGAGTAGAAACATTTTGAAGCTATGGTGTGTGGGGGATCACTTTAATTAATCTATATAACAGTTGAAATTTGGA | This study |
| 3G-2-M-TPLt-TEF1-F | GTCATTTTCGCGTTGAGAAGATGTTCTTATCCAAATTTCAACTGTTATATAGATTAATTAAAGTGATCCCCCACAC | This study |
| 2-M-CYC1t-pEASY -R | CGTATTACAATTCACTGGCCGTCGTTTTACAACGTCGTGACTGGGAAAACCCTGGCGCGTTGGCCGATTCATTAATGC | This study |
| SexAI-IDI1F | CAGACCTGGTATGACTGCCGACAACAATAGTATG | This study |
| AscI-IDI1R | CTCGGCGCGCCTTATAGCATTCTATGAATTTGCCTG | This study |
| SexAI-MAF1F | GACACCTGGTATGAAATTTATTGATGAGCTAGATATAGAG | This study |
| AscI- MAF1R | CGAGGCGCGCCCTACTGTAGGGATTCTTCTTGATCTG | This study |
| ERG20-1-F | ATGGCTTCAGAAAAAGAAATTAGGAGAGAG | This study |
| ERG20-1-R | CGGCGACCAACCAGTAAGCCTGCAACAACTCAATGCACC | This study |
| ERG20-2-F | GTTGCAGGCTTACTGGTTGGTCGCCGATGATATGATGG | This study |
| ERG20-2-R | CTAACATGAATGCGTCCCAGATGGCAATTTCCCCAACTTCAGG | This study |
| ERG20-3-F | GGAAATTGCCATCTGGGACGCATTCATGTTAGAGGCTGC | This study |
| ERG20-3-R | CTATTTGCTTCTCTTGTAAACTTTGTTCAAGAACGC | This study |
| SexAI-LSUF | GACACCTGGTATGAGTGCTCTTGTTAATCCTGTGGCG | This study |
| LSUlinkerR | GGCCATAGAACCACCACCTCAATTGTCCCTATAAGCAATATAATTGGCG | This study |
| linkerSSUF | GGCCATAGAACCACCACCTCAATTGTCCCTATAAGCAATATAATTGGCG | This study |
| AscI- SSUR | CGAGGCGCGCCCTAAGCCGCGTAAAGGCTCGGCT | This study |
| SexAI-GGPS _At_F | GACACCTGGTATGTTATTCACGAGGAGTGTTGCTC | This study |
| AscI- GGPS _At_R | CGAGGCGCGCCTCACTTGTTTCTGGTGATGACTCTATG | This study |

**Table S2 Introduction of alcohol acyltransferases from plants and the titer of geranyl acetate**

| Alcohol acyltransferases | Organism | Titer of geranyl acetate |
| --- | --- | --- |
| SAAT | Fragaria x ananassa | 0.63 mg/L |
| VAAT | Fragaria vesca | 0.10 mg/L |
| AcAAT | Actinidia chinensis | 0 |
| RHAAT | Rosa hybrid cultivar | 0.15 mg/L |
| PHAAT | Petunia hybrida | 0 |

**Fig. S1** Plasmid maps of plant GPP synthase (a) Overexpression of GPPS from *Arabidopsis thaliana* (GPPS_At_); (b) Overexpression of GPPS from *Mentha piperita* (GPPS_Mp_)

_
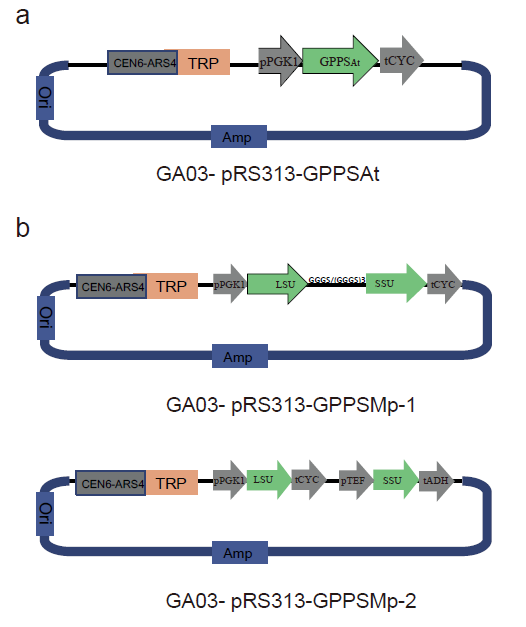
_

**The sequence of optimized gene of SAAT and GES**

**SAAT**

ATGGAAAAAATTGAAGTCTCTATTAATTCAAAACATACAATTAAGCCATCAACATCTTCAACACCATTGCAGCCATATAAATTGACTTTATTGGATCAGTTAACTCCACCAGCTTATGTTCCAATTGTCTTCTTTTATCCAATTACAGATCATGATTTCAATTTGCCACAGACTTTGGCTGACTTGAGACAGGCTTTGTCAGAAACTTTAACTTTGTACTATCCATTATCAGGTAGAGTTAAAAATAATTTGTATATAGACGATTTCGAAGAGGGTGTCCCATACTTGGAGGCTAGGGTCAACTGCGACATGACTGATTTTTTGAGATTAAGAAAGATTGAATGTTTAAACGAATTCGTTCCAATAAAACCATTTTCTATGGAAGCTATATCTGATGAAAGGTATCCATTATTGGGAGTTCAAGTTAATGTTTTTGATTCAGGTATAGCTATTGGTGTTTCTGTTTCTCATAAGTTGATTGATGGTGGT

ACAGCTGATTGTTTTTTGAAGTCTTGGGGTGCTGTTTTCAGGGGTTGTAGGGAAAATATTATTCATCCATCTTTGTCTGAAGCAGCATTGTTGTTCCCACCTAGGGATGACTTGCCAGAAAAATATGTTGATCAAATGGAAGCTTTATGGTTCGCTGGTAAGAAGGTCGCTACTAGAAGGTTTGTCTTCGGTGTTAAAGCTATTTCTTCTATTCAAGACGAGGCTAAGTCTGAGTCTGTCCCTAAGCCATCTAGAGTCCACGCAGTTACTGGTTTTTTGTGGAAACATTTAATAGCTGCATCTAGAGCTTTGACATCTGGTACAACATCTACTAGATTGTCTATTGCAGCTCAGGCTGTCAACTTGAGGACTAGAATGAACATGGAAACAGTTTTAGACAACGCAACTGGTAACTTGTTTTGGTGGGCTCAGGCAATTTTGGAATTGTCTCACACAACACCTGAAATATCTGATTTGAAATTA

TGTGATTTGGTTAACTTGTTAAATGGTTCTGTTAAGCAATGTAACGGTGATTATTTTGAAACTTTTAAAGGTAAGGAAGGATACGGTAGAATGTGTGAATATTTAGATTTCCAGAGAACTATGTCTTCTATGGAGCCAGCACCAGATATTTACTTGTTTTCTTCATGGACAAATTTCTTTAACCCATTGGATTTCGGTTGGGGTAGGACATCTTGGATTGGTGTTGCTGGTAAGATTGAGTCTGCTTCTTGCAAATTCATTATATTGGTCCCAACACAGTGCGGTTCTGGTATTGAGGCATGGGTCAATTTGGAGGAGGAGAAAATGGCTATGTTGGAGCAGGACCCACACTTTTTGGCTTTGGCTTCACCAAAGACATTGATTTAA

**GES**

TCTGCTTGTACTCCTTTGGCATCTGCTATGCCATTGTCTTCTACTCCATTGATTAACGGTGATAATTCTCAGAGAAAAAATACAAGACAACATATGGAAGAGTCTTCATCTAAAAGAAGAGAATATTTGTTGGAAGAAACAACAAGAAAATTGCAAAGAAACGATACTGAATCTGTTGAGAAATTAAAATTGATAGATAACATTCAACAATTAGGTATTGGATACTATTTTGAAGATGCTATAAATGCTGTTTTACGTTCTCCTTTTTCTACAGGTGAAGAAGATTTGTTTACAGCTGCATTGAGATTTAGGTTGTTGAGGCATAATGGTATTGAAATTTCTCCTGAAATTTTCTTGAAGTTCAAAGATGAAAGGGGAAAGTTCGATGAATCTGATACTTTGGGTTTATTGTCTTTATACGAGGCTTCAAACTTGGGTGTTGCTGGTGAAGAGATTTTGGAGGAGGCTATGGAGTTCGCTGAAGCTAGGTTGAGGAGGTCTTTGTCTGAGCCAGCAGCTCCATTGCACGGTGAAGTTGCACAGGCTTTAGACGTCCCAAGGCACTTGAGAATGGCTAGATTGGAAGCTAGAAGATTTATTGAACAATACGGTAAGCAGTCTGACCATGACGGTGACTTGTTGGAATTGGCAATTTTAGATTATAACCAAGTCCAGGCTCAGCACCAGTCTGAATTGACAGAAATTATTAGGTGGTGGAAAGAATTGGGATTGGTTGATAAATTATCTTTTGGTAGAGATAGACCATTGGAATGCTTTTTGTGGACTGTCGGTTTGTTGCCAGAGCCAAAGTATTCTTCTGTTAGAATTGAGTTGGCTAAAGCTATTTCTATTTTGTTGGTTATTGATGATATTTTCGATACATACGGTGAAATGGATGATTTAATTTTGTTCACTGACGCTATTAGAAGGTGGGACTTAGAGGCTATGGAAGGTTTACCTGAATATATGAAGATTTGCTACATGGCTTTGTACAATACTACAAACGAAGTTTGTTATAAAGTTTTAAGAGATACTGGTAGAATTGTTTTGTTAAATTTGAAATCAACTTGGATTGATATGATTGAAGGTTTTATGGAAGAGGCTAAATGGTTCAACGGAGGTTCTGCTCCTAAGTTGGAGGAATATATTGAAAATGGTGTTTCAACTGCTGGTGCTTACATGGCTTTCGCTCATATTTTCTTTTTAATTGGAGAAGGTGTTACTCATCAAAATTCTCAATTGTTCACTCAAAAGCCATATCCAAAAGTCTTTTCTGCTGCAGGTAGAATTTTGAGATTGTGGGACGACTTGGGTACAGCTAAGGAGGAGCAAGAGAGGGGTGATTTAGCTTCTTGTGTTCAATTATTTATGAAGGAAAAATCATTGACTGAAGAGGAGGCTAGATCTAGAATTTTGGAAGAAATTAAGGGTTTATGGAGAGATTTGAATGGTGAGTTGGTCTATAATAAGAATTTACCATTATCAATTATTAAAGTCGCTTTGAACATGGCTAGGGCTTCTCAGGTCGTCTATAAACACGATCAAGATACTTACTTTTCTTCTGTTGACAATTACGTTGATGCTTTATTTTTCACTCAATAA
